# Evolution of Volatile Aroma Compounds and Amino Acids in Cabernet Gernischt Grape Berries (*Vitis vinifera* L.): Comparison of Different Training Systems for Mechanical Soil Burial

**DOI:** 10.3390/foods11111568

**Published:** 2022-05-26

**Authors:** Kangqi Geng, Dongmei Li, Jing Zhang, Yanxia Zhang, Zhennan Zhan, Zhenping Wang

**Affiliations:** 1 School of Life Sciences, Ningxia University, Yinchuan 750021, China; gengkangqi@163.com (K.G.); zhangyanxiaedu@163.com (Y.Z.); znzhan@126.com (Z.Z.); 2 College of Agriculture, Ningxia University, Yinchuan 750021, China; ldm651011@sjtu.edu.cn (D.L.); zj627202@126.com (J.Z.)

**Keywords:** Cabernet Gernischt, training systems, volatile aroma compounds, amino acids, gene expression

## Abstract

Soil burial is a survival strategy for grapevines that can safely overwinter in north-western regions of China. A suitable training system was beneficial for soil burial to provide winter protection. Moreover, the training system can also significantly affect fruit quality during the development of grape berries, such as primary and secondary metabolites. In this study, four-year-old Cabernet Gernischt grapevines were used as experimental material and exposed to four training systems, including the Ningxia traditional vertical trunk (control, CK); the gobelet (T1); single guyot (T2); slant trunk with vertical shoot positioning (STVSP) (T3). The results showed that total soluble solid total phenol content was 12.69%, 57% higher under T3 training systems than in the control, and T3 alleviated the canopy density, leading to improving the leaf photosynthetic efficiency gas chromatography-mass spectrometry (GC-MS) assay used to detect the aroma compounds. The results indicated that the T3 training system enhanced the accumulation of alcohols, carbonyl compounds, C6/C9 and esters, which account for the largest proportion of volatile compounds, and the qRT-PCR reveals that *VvEcar*, *Vvter*, *VvCCD1*, and *VvLis* were raised under T3 at the transcriptional level. Moreover, T3 contributes to most free amino acid synthesis. Additionally, the PCA reveals the correlation of free amino acids under four training systems, which reflected the mostly amino acid related to T3, and thus, we could speculate that T3 enhances the overall aroma. These results may lead to new strategies to select a new, short trunk training system to achieve mechanized buried soil, to prevent cold and produce high-quality wine in this area.

## 1. Introduction

Grape berry development and ripening are dynamic processes that involve a complex series of biochemical changes [1]. Aroma compounds are secondary metabolites that play a key role in grape quality for enological purposes. Terpenes, C_13_-norisoprenoids, phenols, and non-terpenic alcohols are the most important aroma compounds in grapes, and can be found as free volatile or glycol-conjugated (bound) molecules [2]. Previous reports have shown that more than 900 kinds of volatile aroma substances were identified and isolated from grape wine, and furthermore, the complexity of wine aroma can also vary depending on many variables, such as the type of wine, grape variety, terroir, microbial starter, fermentation process, aging, and bottling [3]. Among them, the variety characteristic aroma plays a decisive role. The content of volatile aroma compounds changes during the process of grape berry ripening, for instance, varietal volatile compounds content reached a maximum at maturity, as established by the sugar/acidity ratio, and remained constant in the following weeks in the red grapes [4]. In ‘Cabernet Sauvignon’, esters are characteristic of early berry development, aldehydes of middle development stages, and alcohols of late development [5]; moreover, terpenes (predominantly eucalyptol, *β*-caryophyllene, and *R*-humulene) are prevalent during early development, while benzene derivatives such as 2-phenylethanol and 2-phenylethanal tend to appear later. The late dominance of alcohols is desirable, because alcohols usually have not only higher herbaceous odor thresholds than related aldehydes, but also a greater propensity to form fruity esters in the presence of carboxylic acids during vinification [6,7]. Recently, the genes of key enzyme in the anabolic pathway of terpenoids have been verified; for instance, (*E*)-*β*-caryophyllene synthase was encoded by *VvEcar*; in addition, *VvLIS*, *VvCCD1* and *Vvter* are also involved in the terpenoid synthesis pathway [8].

Choosing an appropriate training system is based primarily on growth habits and the need for optimum fruit exposure, and is affected by vine vigor and winter hardiness. Adopting a reasonable training system according to the local “Terroir” could modify the microenvironment of the canopy by changing the temperature, humidity, and radiation, thereby achieving balanced vegetative growth and reproductive growth, which is beneficial to the improvement of fruit quality (sugars, acids, anthocyanins) [9]. The east foot of Helan Mountain in Ningxia (China) is known as one of the best wine-producing regions, but many grape growers did not adopt a standard training system to purse a higher yield at the beginning of vineyard construction; otherwise, this region has a continental semi-arid climate, and the extreme temperature in winter is below −22 °C. In buried soil for winterization, choosing a training system should also consider the convenience of burying the vines into soil.

Different training systems influence the berry volatile component. Flor Etchebarne [10] compared Viognier training with vertical shoot positioning (VSP) and the minimal pruning (MP) system, and concluded that the MP training system provided a greater aroma potential for grapes than the VSP system, due to the positive effect on the concentration of aroma glycoside precursors by a reduction in berry size. Li [11] showed that crawled cordon training (CCT) improved the accumulation of aroma compounds in early harvesting time; if harvesting time needs to be postponed, independent long-stem pruning (ILSP) was also a suitable selection. ‘Cabernet Gernischt’ (*Vitis vinifera* L. cv.) is the most important red wine cultivar in China. However, there are few works published on volatile compounds relative to this variety. In 2008, Li et al. established that ‘Cabernet Gernischt’ is in fact ‘Carmenère’ rather than the previous ‘Cabernet Franc’ [12]; it belongs to neutral varieties (low or no concentration of free monoterpenes) [13] and usually has fresh, fruit flavors, and potentially leafy, vegetal notes.

In order to facilitate burying in winter and pursue high yields, the grapevines are mostly shaped in the form of crawled cordon training in the burying soil area. As the vine trunk must be put flat before burying soil, which is labor-intensive and time-consuming, it will cause about 2% to 5% of the vine damage each year. In addition, these traditional types of training could lead to different fruiting zones, making it difficult to produce quality wine and mechanized operation. In recent years, with the increase of labor costs, a simple off-shelf method is needed to reduce production costs and meet the stability of grape quality. Thus, this study was conducted over four short trunk training systems on ‘Cabernet Gernischt’ to compare the accumulation of volatile flavor substances and the expression of related genes during grape berry ripening. It is expected to select a suitable cultivation method in the off-shelf area, which is conducive to improving the volatile flavor substances in grape berries for production, and assisting the viticulturer in grasping theoretical guidance for scientific cultivation and management.

## 2. Materials and Methods

### 2.1. Vineyard Description and Berry Sampling

This study was conducted with 4-year-old own-rooted red wine grapevines ‘Cabernet Gernischt’ (*Vitis vinifera* L.), which were harvested in the 2016 vintage from Yuquanying (YQY) Farm of Ningxia Autonomous Region, China (38°14′19″ N, 106°2′0″ E). Vines were oriented in the east-west direction with drip irrigation (2.8 L/h, irrigation time determined by the water consumption). The vines were spaced at 3.0 × 1.0 m, the soil was Aeolian sandy soil, the fertility was medium, and pH was 8.2 (Basic physical and chemical properties of Aeolian soil were presented in Table S1).

**Figure 1:**
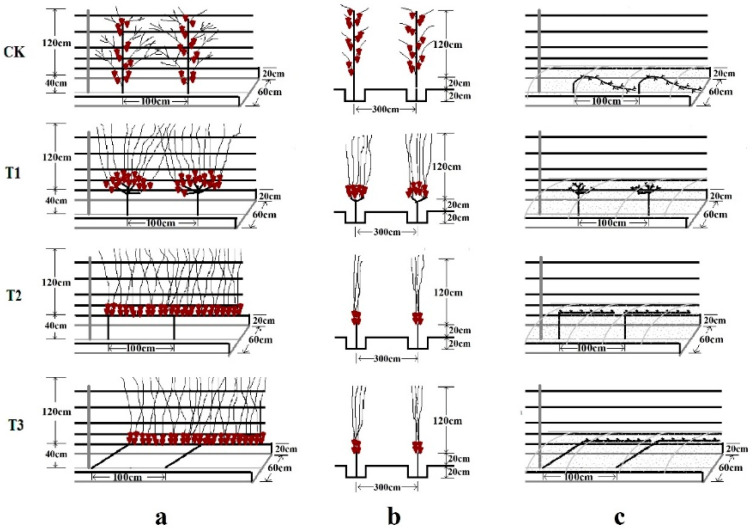
The Ningxia traditional vertical trunk used as control (CK); the gobelet (T1); single guyot (T2); slant trunk with vertical shoot positioning (STVSP) (T3). (**a**,**b**) indicated the positive and side design sketch, (**c**) indicated the phenotype in winter.

This field experiment was designed with a single-factor randomized complete block with three replicates of 6 rows and 100 vines planted in each row (the middle 4 rows were sampling areas, and the two sides were buffers). Four different training systems adapted to the constraints of Ningxia were compared: the Ningxia traditional vertical trunk was used as control (CK); the gobelet (T1); single guyot (T2); slant trunk with vertical shoot positioning (STVSP) (T3), the detailed technical operation was presented in Figure 1 and Table S2. Pest management, irrigation, and fertilization were conducted according to the standard agronomic practices of the region.

For each training system, 300 berries as 3 biological replicates were randomly collected from more than 60 vines on both sides of the canopy from pre-veraison to maturity (50, 65, 70, 90, and 120 DAA), frozen in liquid nitrogen immediately, and stored at −80 °C until analyses.

### 2.2. Measurement of Canopy Microenvironment

The temperature and humidity were measured by Lascar Electronics Ltd., Erie, PA, USA.

### 2.3. Measurement of Canopy Photosynthetic Efficiency

The net photosynthetic rate (Pn) and transpiration rate (Tr) were measured with the TP-3051D photosynthetic apparatus (Zhejiang Top Instrument Co., Ltd., Hangzhou, China).

### 2.4. Measurement of Berry Quality

Prior to analyzing berry quality, these 300 berries were used to weigh and calculate the one hundred berry weight. Total soluble solid and titratable acidity were measured in the juice obtained by crushing the berries of each replicate, and meanwhile, the total soluble solid content was determined using a TD-45 digital refractometer (Zhejiang Top Instrument Co., Ltd., Hangzhou, China). Titratable acidity was measured by titration with 0.1 mol/L NaOH and expressed in g/L [14]. Total phenols were measured by Folin–Ciocalteu and expressed in mg/g [15].

### 2.5. Extraction and Determination of Volatile Compounds

#### 2.5.1. Isolation of Aroma Compounds

Liquid nitrogen was used to grind frozen grape berries (10.0 g) into powder and transfer them to a 50 mL centrifuge tube; 0.5 g of D-gluconolactone and 1 g of cross-linked polyethylene poly-pyrrolidone (clarifying agent) were added to the centrifuge tube; after a brief vortex, the mix was incubated for 120 min at 4 °C; the clarified grape juice was obtained by centrifugation at 9000× *g* and 4 °C for 10 min. Each treatment was repeated three times.

#### 2.5.2. Headspace Solid-Phase Microextraction

Moreover, 5 mL clarified grape juice, 1 g NaCl, 5 μL internal standard 2-octanol (0.5%), and a magnetic rotor were put into a 15 mL sample bottle. We tightened the lid of the top empty bottle quickly, and placed it on the magnetic stirring heating table, when an activated 75 μM polydimethylsiloxane/carbon sieve/diethyl benzene (PDMS/CAR/DVB) extraction head was inserted into the headspace bottle, 1 cm above liquid level. After stirring and heating at 37 °C for 40 min, the volatile substances in the headspace bottle reached an equilibrium state. Then, the extraction head was removed from the sample bottle and inserted into the gas chromatographic inlet, and the sample was resolved at 250 °C for 3 min.

#### 2.5.3. GC-MS Analysis

The gas chromatography-mass spectrometer was GCMS-QP2010 (Shimadzu Co., Kyoto, Japan); the chromatographic column was HP-5MS (30 m × 0.25 mm × 0.25 μm) [16]. Helium was selected as the carrier gas, and flowed at a constant rate of 0.8 mL/min in spitless mode. The oven temperature was programmed to remain at 35 °C for 3 min and then increase to 120 °C at 4 °C/min, where it was held for 2 min. The injection temperature was set to 250 °C. The MS conditions were as follows: electron ionization (EI) mode at 70 eV; 200 °C ion source temperature; ionization mode EI, detector power supply 350 eV.

#### 2.5.4. Qualitative and Relative Quantitative Analysis

The identification of the volatile compounds was based on the retention indices (RIs) of reference standards and mass spectra, matching the standard NIST 98 MS database [17]. The internal standard method was used for relative quantification (assuming that the correction factor was 1), and the peak area of the internal standard was compared with the peak area of each component to calculate the content of volatile aroma substances relative to the internal standard [18].

### 2.6. Determination of Amino Acid Content

#### 2.6.1. Sample Preparation

We followed the methodology of the previous study, with slight modifications [19]. We prepared a 6 mol·L^−1^ hydrochloric acid solution containing 1% phenol. The samples of cereals (CP 10–25%) 100 mg, cake concentrate (CP 30%) 80 mg, fish meal protein meal (CP 50–65%) 60 mg, feather meal and blood meal (CP 70–80%) 50 mg were weighed. We transferred the weighed sample into the digestion tube, blew nitrogen for 2 min to replace the internal air, and sealed it. The tube was hydrolyzed for 22–24 h in a drying oven at 110 °C. After hydrolyzation, we transferred it to a 50 mL volumetric flask with deionized water. We took 300 μL into a 1.5 mL centrifuge tube and blew it dry with nitrogen. We added 1 mL 0.02 mol·L^−1^ hydrochloric acid solution to the centrifuge tube and dissolved it via ultrasonic. It was centrifuged at 3000× *g* for 3 min after dissolving. The supernatant was filtered by 0.45 μm aqueous phase membrane before determination.

#### 2.6.2. Content Determination

The determination of free amino acids in berries was carried on an automatic Amino Acid Analyzer (HITACHIL-8900, Amino Acid Analyzer, Tokyo, Japan). The chromatographic column was 4.6 mm × 40 mm, with sulfonated cationic resin; the flow rate was 0.999 mL/min, the detection wavelengths were 570 nm and 440 nm; the injection volume was 20 μL.

Reduction formula

Content (%) = X × (M/m) × (25/3) × 10^−4^

X was measured value, M was molar mass (g/mol), m was sample weight (g).

### 2.7. Determination of Genes Involved in Flavour Formation in Grape Berries

The total RNA was extracted by using the PEXBIO Plant Fruit Kit (Beijing APEXBIO Biotechnology, Beijing, China) according to the manufacturer’s instructions. The purity and integrity of RNA were analyzed by Nanodrop 2000 (Thermo Fisher Scientific, Waltham, MA, USA). cDNA was synthesized by using 1 µL of total RNA as a template using Takara PrimeScript™ RT reagent Kit with gDNA Eraser (Takara Biomedical Technology, Beijing, China). Reverse transcription dilution can be used directly for quantitative PCR. Actin (Accession No. EC969944) was selected as a reference to calculate expression profiles, primers sequences of *VvEcar*, *VvCCD1*, *Vvter* and *VvLIS* used for quantitative real-time PCR (RT-qPCR) were designed according to Genebank (http://www.idtdna.com/primerquest/Home/Index accessed on 1 May 2022) and listed in Table S3.

RT-qPCR was performed on a qTOWER 2.0 PCR detection system (Analytik Jena AG, Jena, Germany). The reaction mixture (25 µL) contained a1 µL cDNA template, 0.5 µL-specific forward, and reverse primers to each gene, 12.5 µL 2 × UltraSYBR Mixture (CWBIO), and 10.5 µL ddH_2_O. Thermal cycling conditions were as follows: 95 °C for 10 min followed by 95 °C for 15 s, 60 °C for 1 min under 40 cycles. Fluorescence was collected in the second step, and the ddH_2_O was used to replace the cDNA as the control. Each sample was run in three replicates. Gene transcripts were quantified upon normalization to Actin gene by comparing the threshold cycle (Ct) of each target gene with the geometric mean of Actin and EF Ct. The relative quantification per each gene was calculated by the 2^−ΔΔct^ method [20].

### 2.8. Statistical Analysis

SPSS 20.0 (IBM, Armonk, NY, USA) was used to analyze the statistical parameters. A one-way analysis of variance (ANOVA) and an LSD test were conducted to detect significant differences at *p* ≤ 0.05. The plots were prepared using Origin 9.0 (OriginLab Corporation, Northampton, MA, USA) and R for windows (4.0.5). A principal component analysis (PCA) was undertaken on all significantly different variables after standardization using The Unscrambler X (CAMO Software, Oslo, Norway). All samples were repeated 3 times, and data were present with mean ± sd.

## 3. Results and Discussion

### 3.1. Canopy Microclimate and Photosynthesis Index under Four Training System

The training system is an essential factor to manage the canopy, which plays a fundamental role in light energy capture via photosynthesis, as it regulates transpiration and microclimate of ripening grapes, thus affecting yield and quality [21]. So, we measured the temperature, humidity, Pn, and Tr of the canopy after sampling. The temperature and humidity could reflect the degree of exposure; as reported in Table 1, the temperature and humidity under CK were always kept lowest and highest, basically. The Ningxia traditional vertical trunk training system is caused by pursuing the high yield which usually causes the canopy to close. This aggravated the frequent occurrence of pests and diseases and low-quality grape berries. Relatively speaking, the T2 and T3 treatments could retain a higher temperature and lower humidity; this shows that this training system could receive more light so as to biosynthesize more metabolisms.

Net photosynthesis rate (Pn) and transpiration rate (Tr) on behalf of the leaf photosynthesis ability and stomatal conductance. As shown in Table 2, the Pn under CK treatment is obviously lower than the other training systems. Accordingly, the Tr under T2 treatment acts out the lowest value, thus leading to an overall reduction in water consumption. Thus, the results are consistent with previous studies, which is the lower number of nodes along the cane and their better spatial and light distribution [9].

### 3.2. Basic Physicochemical Parameters of Berry Development

**Figure 2:**
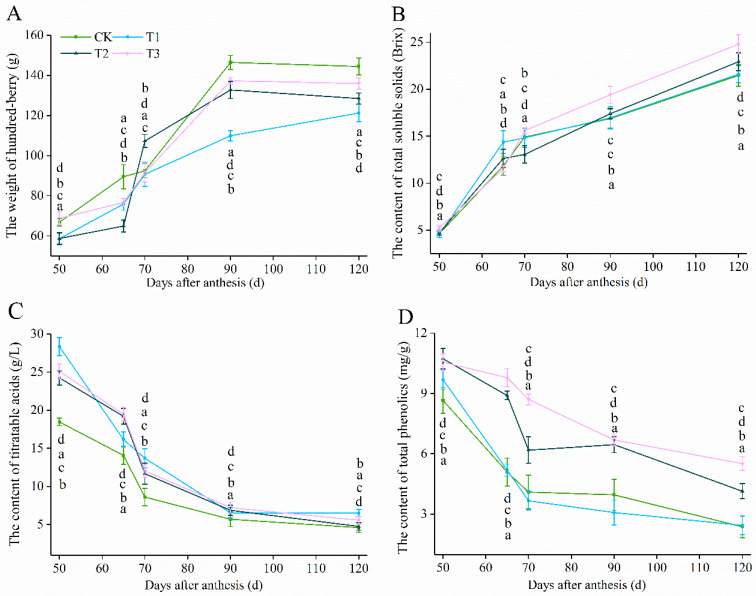
Physical characteristics of differently training system of ‘Cabernet Gernischt’ (*Vitis vinifera* L.) berries in 2016. Overview the changes of hundred-berry weight (**A**), total soluble solids (**B**), titratable acids (**C**) and total phenolics (**D**) during developmental stages, where error bars represent the SD. Different letters denote significant differences separately for individual factors by Tukey’s test at *p* < 0.05 (From top to bottom represents the CK, T1, T2, T3). The details were summarized in Table S4.

The data from quantitative analysis of hundred-berry weight, total soluble solids (TSSs), titratable acidity (TA) and total phenols are shown in Figure 2A–D and Table S4. The hundred berry weight increased rapidly after veraison and kept a relatively stable state at 90 DAA; among all treatments, CK was significantly higher than other treatments at harvest. Meanwhile, CK had the lowest total soluble solids, owing to the productivity at maturity, while T3 kept the highest Brix at harvest. The amount of total phenolics decreased rapidly from 65–70 DAA and was maintained stably after 90 DAA. The changes of titratable acids showed a significant difference between T2, T3 with CK, and T1. As we can see from Figure 2D, CK and T1 decreased sharply from 50–70 DAA, CK, and T1 to keep a higher content of titratable acids at maturity. The results of this study indicate that different training systems had significant effects on fruit quality, and therefore it will have a significant influence on secondary metabolism and subsequent fermentation [22,23].

### 3.3. Aroma Components Analysis

**Figure 3:**
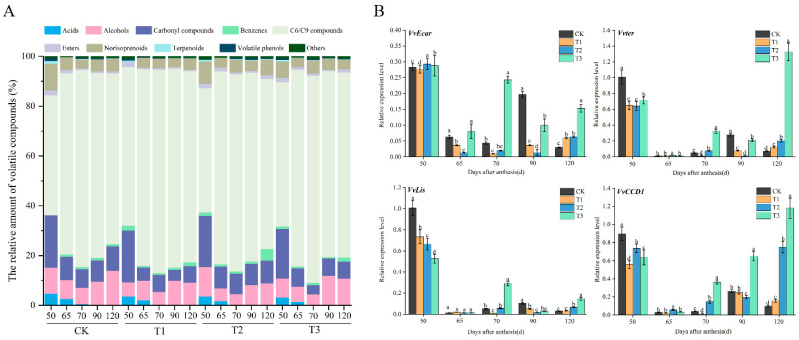
(**A**) Percentage stacked bar chart showing the volatile compounds of the different training system of ‘Cabernet Gernischt’ (*Vitis vinifera* L.) during grape maturity. (**B**) Four aromas related genes under the different training system of ‘Cabernet Gernischt’ *(Vitis vinifera* L.). Different letters denote significant differences separately for individual factors by Tukey’s test at *p* < 0.05.

The volatile compounds in each developmental stage are shown in Figure 3A. In this experiment, a total of 62, 62, 61, 60 volatile compounds were identified from CK, T1, T2, and T3 training systems, respectively (Table S5). We classify these volatile compounds in grape berry into ten kinds, and C6/C9 compounds kept the most abundant free compounds during all stages; the C6/C9 compounds are synthesized by the LOX and HPL synthetic pathway, and mainly present ‘grassy’ and ‘green’ flavors [24], as shown in Figure 3A. The contents of C6/C9 compounds rose after 65DAA and turned to a downward trend immediately; this is because the sugar began to accumulate concurrently, and the free volatile compounds transformed into bound volatile compounds under glycosylation, but some researchers have indicated that volatile evolution is not proportional to the changes in the sugar content of grapes in neutral varieties [25]; otherwise, in the LOX pathway of ‘Shine Muscat’, LOX, ADH, and substrate alcohols were the limiting factors for the corresponding reactions [26]. Curiously, the C6/C9 compounds under T3 treatment maintain the highest content at harvest, which is 2253.67 μg/L. We conjecture that the canopy temperature and microclimate were better in T3 treatment (high light/low humidity). The second-highest content compounds are carbonyl compounds, which shows rising with the development of berries. Among them, the T2 treatment has the highest content at harvest. The higher alcohols exhibited the highest content under T3 treatment at harvest, and the rate of conversion from aldehyde to alcohol is strongly related to the activities of these enzymes, which is dependent on variety or Vitis species [27]. Norisoprenoids and terpenoids had low concentrations in Cabernet Gernischt berries in the whole growth period, but they make important contributions to the characteristic varietal aroma, owing to their extremely low odour thresholds [28]. Compared with other fruits, the aroma characteristics of grape berries are characterized by terpenes, especially monoterpenes, which primarily result in a rich rose aroma, and sharply decreased from the fruit-set to the veraison period [26]. Similar results were found in this study; linalool, *α*-terpineol, *β*-citronellol, geraniol, and myrcene belong to monoterpenes, and the contents of terpenes increase slightly during the growth period. However, in non-aromatic grapes, what is more important is the reduction of isoprene substances, which are also characterized by flower and fruit aromas. Temperature is a key factor affecting the physiology of wine grapes, which have regulation effects on C_13_-norisoprenoids via the influence of the cycle growth of the grape [29]. It was found that, under the condition of higher temperature, the contents of *β*-damascone, *β*-ionone, and *α*-ionone in wine were higher [30], so that the high temperature during the growth period and the large temperature difference between day and night in Ningxia may be the reasons for the high content of C_13_ in this area. Moreover, the T1 and T2 treatments contain more C_13_-norisoprenoids; we speculate that leaves can accept more light under this treatment, which result in changes to the canopy microclimate [31].

For *VvEcar* involved terpenoid synthesis, its present highest level is 50 DAA and declines after veraison, and it gradually becomes higher at 120 DAA. *Vvter* regulates *α*-terpineol synthase expression at the transcriptional level; similar to *VvCCD1*, the expression level is the lowest at veraison, and tends to rise from 70 DAA to 120 DAA; the T3 treatment was kept at its highest level at harvest. *VvLis* is a key gene in the late phase of the monoterpene anabolic pathway; the expression level is the highest at young grape berries and rapid decline, otherwise, it rises at 70DAA and the T3 treatment is kept at its highest level. *VvCCD1* regulate the degradation of carotenoids to produce C_13_- isoprene derivatives, the expression level of *VvCCD1* is highest at 50 DAA, in turn, it decreased to minimum level at 65 DAA. After veraison, the expression level began to uptrend for each treatment, among the T2 and T3, which was significantly higher than CK and T1.

### 3.4. Free Amino Acid Profiles of Grape Berries

Amino acids were linked with fruit aroma by acting as precursors for the biosynthesis of aroma-forming volatile compounds. Table 3 showed the free amino acid profiles from CK and other treated vines by regulating the training system. A total of 15 types of free amino acids were detected in the grape berries. Glu, Ala, Val, Pro, and Phe were the most abundant amino acids in all the grape berries. Glu is preferably utilized by Saccharomyces cerevisiae during fermentation. Val can be converted into isobutanol, propanol, isoamyl alcohol, and active amyl alcohol in the process of wine brewing. These higher alcohols can also be further formed into higher alcohols acetic ester, which are the main fermented flavor components in wine. Pro is a plant compatible osmotic agent, which is beneficial to resist osmotic stress [32]. Phenylalanine is an *α*-amino acid and a substrate of the phenylalanine ammonia lyase enzyme (PAL) that catalyzes the conversion of phenylalanine into cinnamic acid as the first step of the biosynthesis of plant phenolic compounds [33]. In addition, certain volatile compounds such as 2-phenylethanol and 2-phenylethyl acetate are formed by this aromatic amino acid due to the enzymatic activity of yeast, which contributes to the floral wine aroma [34]. In terms of above five types and total free amino acids, T3 treatment could significantly increase the content at harvest (*p* < 0.05).

### 3.5. The Correlation Analysis between Amino Acids in Grape Berries

**Figure 4:**
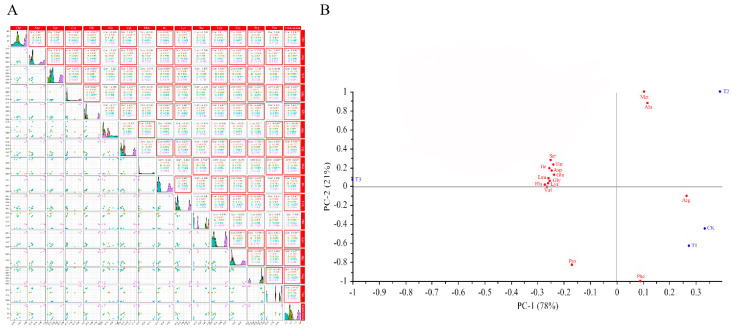
(**A**) Correlation analysis of the contents of amino acids in ‘Cabernet Gernischt’ (*Vitis vinifera* L.) with different treatments. The amino acids with relevant significance were marked in the red box (*p* ≤ 0.05). (**B**) 15 amino acids on PC1 vs. PC2 by bi-plot (scores plot and loadings plot).

The difference of amino acid content in wine grape will affect the fermentation speed; Arg, Ala, and Glu are important nitrogen sources of Saccharomyces cerevisiae, while Pro and Thr are difficult to be utilized by yeast [35]. However, the low content of Arg in this study may be related to the soil type in this region. The ethyl propionate, isobutanol, acetic acid, and octanoic acid in wine aroma components can be expressed by multiple regression model with eight amino acids: Asp, Thr, His, Phe, Ser, Pro, Arg, and Met [34]. A correlation analysis of amino acids showed that Asp was positively correlated with Thr, His, Ser, and Pro, and negatively correlated with Arg significantly under CK treatment (Figure 4A). There was a significant positive correlation between Thr, His, Ser, Arg and Pro under T1 treatment; Phe was negatively correlated with Arg and Met. There was a significant positive correlation between Ser under CK, T1, and T2 treatments. Pro, and Ser were negatively correlated with Arg. There was a significant negative correlation between Pro and Arg and Met under T3 treatment. Therefore, this region should supply nitrogen fertilizers after vineyard regulation to improve the content of Arg. Many previous studies have reported that the changes in amino acid content in grape can affect wine volatile composition [36], but rarely consider the correlation between the training systems with free amino acids. Or say, the training system could alter the delivery value or change the amount of synthetics, and therefore, it can provide a theoretical basis for fertilization in this area, so as to increase the content of volatile aroma in grape berry.

Figure 4B shows that the principal components (PC) contribution rate of 15 amino acids compound cumulative variance is 99%, representing 78% and 21% of the total attributable to PC 1 and PC 2, respectively. The distribution of different treatments is more scattered, CK and T1 were located in right bottom quadrant and had highly relevance with Arg and Phe; T2 was located in right bottom quadrant and had high relevance with Met and Ala; T3 was located in the left bottom quadrant, which was associated with mostly amino acids. According to the previous study, the mainly aroma-producing and flavoring-producing substances in Cabernet Gernischt are (*Z*)-3-hexen-1-ol, *β*-damascenone, *β*-ionone, (*E, Z*)-2,6-nonadienal, 4-terpineol, *α*-terpineol, benzyl alcohol, 2-phenylethanol, and phenol [37]. The above substances will impart the unique floral and grassy aroma, and we often think that grassy is the typical variety aroma of Cabernet gernisch. Otherwise, Zhu et al. found that alanine, phenylalanine, and isoleucine have the potential to increase fruity ester production [38]; nevertheless, in our results, the Ala close to T2 and Arg was close to CK, so we speculate that CK has a higher density conapy, which caused insufficient berry ripening, so that they have a more green and glassy odor. As for T3, although mostly amino acids could not fortify variety aroma on the basis of present studies, it is quite important to increase the overall aroma.

## 4. Conclusions

Four training systems were compared in Ningxia to explore the new training system, which influenced berry quality, volatile aroma, and free amino acid during the whole grape berry development period. The results showed that the hundred berry weight of T3 was the smallest; the soluble solids and total phenol contents were 12.69% and 57% higher than CK, which met the requirements of making high-quality wine. Moreover, 62, 62, 61, and 60 kinds of volatile compounds were identified from CK, T1, T2, and T3 training systems, respectively, whereas T3 has the highest content (up to 3035.254 mg/L). Thr, Asp, Ser, Glu, Gly, Val, Ile, Leu, Lys, His, and Pro were the highest under T3 and significantly higher than CK, among the contents of Glu, was the highest, accounting for 18.2% of the total free amino acids. Overall, the fruit quality under T3 treatment can better meet the standard of making high-quality wine, and it has a good accumulation effect on the amino acids and aroma substances during the berry development process, in terms of actual production, it has more convenience for descending from the shelf and bury soil for overwinter.

## Figures and Tables

**Table 1:** The temperature and humidity under four training systems.

	Temperature (°C)					Humidity (%)				
	50	65	70	90	120	50	65	70	90	120
CK	21.53 ± 1.27 d	25.42 ± 2.12 d	23.77 ± 1.48 d	24.03 ± 1.46 d	23.14 ± 1.63 c	38.1 ± 0.8 a	34.8 ± 1.6 a	35.4 ± 1.2 a	37.2 ± 1.6 a	36.2 ± 2.1 a
T1	22.18 ± 1.53 c	25.77 ± 1.76 c	23.89 ± 1.23 c	24.71 ± 2.11 c	22.07 ± 1.68 d	36.4 ± 1.3 c	33.2 ± 0.8 c	32.4 ± 0.7 b	36.5 ± 0.5 b	34.1 ± 1.3 c
T2	22.42 ± 2.05 a	26.97 ± 2.83 b	24.82 ± 2.14 a	25.24 ± 1.35 a	24.19 ± 1.43 b	37.2 ± 0.9 b	33.7 ± 1.4 b	32.1 ± 1.7 b	35.8 ± 1.0 c	34.6 ± 1.8 b
T3	22.37 ± 1.88 b	27.15 ± 2.35 a	24.18 ± 3.26 b	25.12 ± 1.52 b	24.34 ± 1.67 a	33.8 ± 1.1 d	31.3 ± 0.7 d	30.7 ± 1.5 c	34.7 ± 0.4 d	33.6 ± 0.9 d

Note: All data were measured at 11 o’clock after sampling, data represent the average (±sd) of *n* = 3 biological replicates, each with three technical repetitions. Small letters represent the significant differences at *p* ≤ 0.05.

**Table 2:** The Pn and Tr under four training system.

	Pn (μmol CO_2_·m^−2^·s^−1^)					Tr (mol H_2_O·m^−2^·s^−1^)				
	50	65	70	90	120	50	65	70	90	120
CK	6.45 ± 0.58 c	6.97 ± 0.83 d	7.05 ± 0.43 d	6.79 ± 0.82 d	6.55 ± 0.46 d	0.6 ± 0.11 a	0.8 ± 0.13 a	0.7 ± 0.12 a	0.8 ± 0.17 a	0.6 ± 0.05 a
T1	7.11 ± 0.73 b	7.74 ± 0.58 c	7.57 ± 0.51 c	7.98 ± 0.57 c	7.19 ± 0.48 c	0.3 ± 0.07 a	0.4 ± 0.06 ab	0.6 ± 0.14 a	0.7 ± 0.07 a	0.6 ± 0.03 a
T2	7.52 ± 0.77 a	8.21 ± 0.53 b	8.06 ± 0.78 b	8.43 ± 0.32 b	8.31 ± 0.47 a	0.4 ± 0.08 a	0.4 ± 0.05 ab	0.7 ± 0.11 a	0.6 ± 0.15 a	0.5 ± 0.03 a
T3	7.49 ± 0.82 a	8.34 ± 0.45 a	8.13 ± 0.33 a	8.72 ± 0.72 a	7.56 ± 0.66 b	0.3 ± 0.04 a	0.3 ± 0.07 b	0.4 ± 0.05 a	0.6 ± 0.13 a	0.5 ± 0.06 a

Note: All data were measured at 11 o’clock after sampling, data represent the average (±sd) of *n* = 3 biological replicates, each with three technical repetitions. Small letters represent the significant differences at *p* ≤ 0.05.

**Table 3:** Free amino acid content (%) in grape berries at harvest under different training systems. All parameters are performed with their standard deviation (*n* = 3). For each parameter, with the same column, different letters indicate significant differences between treatments by Tukey HSD (*p* ≤ 0.05).

Free Amino Acid	CK	T1	T2	T3
Asp	0.038 ± 0.003 b	0.045 ± 0.014 b	0.044 ± 0.003 b	0.099 ± 0.002 a
Glu	0.1 ± 0.006 b	0.094 ± 0.005 b	0.1 ± 0.008 b	0.289 ± 0.007 a
Ser	0.044 ± 0.002 b	0.041 ± 0.003 b	0.047 ± 0.002 b	0.08 ± 0.001 a
His	0.037 ± 0.001 b	0.038 ± 0.003 b	0.037 ± 0.002 b	0.058 ± 0.004 a
Gly	0.025 ± 0.004 b	0.036 ± 0.007 b	0.032 ± 0.003 b	0.097 ± 0.003 a
Thr	0.04 ± 0.002 b	0.035 ± 0.003 b	0.039 ± 0.002 b	0.061 ± 0.002 a
Arg	0.086 ± 0.002 a	0.08 ± 0.001 b	0.081 ± 0.001 b	0.037 ± 0.003 c
Ala	0.225 ± 0.05 b	0.207 ± 0.006 c	0.261 ± 0.03 a	0.212 ± 0.001 c
Val	0.09 ± 0.003 bc	0.086 ± 0.004 c	0.085 ± 0.002 c	0.123 ± 0.001 a
Met	0	0	0.057 ± 0.005 a	0
Lys	0.048 ± 0.001 b	0.045 ± 0.003 b	0.046 ± 0.004 b	0.072 ± 0.001 a
Leu	0.028 ± 0.002 b	0.032 ± 0.005 b	0.031 ± 0.004 b	0.075 ± 0.001 a
Pro	0.128 ± 0.004 a	0.169 ± 0.003 a	0.04 ± 0.001 b	0.188 ± 0.04 a
Ile	0.012 ± 0.003 b	0.014 ± 0.002 b	0.014 ± 0.001 b	0.036 ± 0.001 a
Phe	0.299 ± 0.001 a	0.266 ± 0.007 ab	0.122 ± 0.006 c	0.165 ± 0.07 bc
Total free amino acid	1.2 ± 0.06 b	1.191 ± 0.01 b	1.036 ± 0.03 b	1.592 ± 0.14 a

## Data Availability

No new data were created or analyzed in this study. Data sharing is not applicable to this article.

## Supplementary Materials

The following supporting information can be downloaded at: https://www.mdpi.com/article/10.3390/foods11111568/s1, Table S1: Basic physical and Chemical Properties of Aeolian soil; Table S2: Technological details of four training system; Table S3: Primer sequences of real-time fluorescence quantitative PCR; Table S4: Physical characteristics of differently training system of ‘Cabernet Gernischt’ (*Vitis vinifera* L. cv.) berries in 2016; Table S5: Concentrations (μg/L, mean ± SD) of volatile aroma compounds during grape development under different training system.

## References

[1] Lombardo V.A., Osorio S., Borsani J., Lauxmann M.A., Bustamante C.A., Budde C.O., Andreo C.S., Lara M.V., Fernie A.R., Drincovich M.F. (2011). Metabolic Profiling during Peach Fruit Development and Ripening Reveals the Metabolic Networks That Underpin Each Developmental Stage. Plant Physiol..

[2] Alem H., Rigou P., Schneider R., Ojeda H., Torregrosa L. (2019). Impact of Agronomic Practices on Grape Aroma Composition: A Review. J. Sci. Food Agric..

[3] Ruiz J., Kiene F., Belda I., Fracassetti D., Marquina D., Navascués E., Calderón F., Benito A., Rauhut D., Santos A. (2019). Effects on Varietal Aromas during Wine Making: A Review of the Impact of Varietal Aromas on the Flavor of Wine. Appl. Microbiol. Biotechnol..

[4] Coelho E., Rocha S.M., Delgadillo I., Coimbra M.A. (2006). Headspace-SPME Applied to Varietal Volatile Components Evolution during *Vitis vinifera* L. Cv. ‘Baga’ Ripening. Anal. Chim. Acta.

[5] Kalua C.M., Boss P.K. (2009). Evolution of Volatile Compounds during the Development of Cabernet Sauvignon Grapes (*Vitis vinifera* L.). J. Agric. Food Chem..

[6] González-Barreiro C., Rial-Otero R., Cancho-Grande B., Simal-Gándara J. (2015). Wine Aroma Compounds in Grapes: A Critical Review. Crit. Rev. Food Sci. Nutr..

[7] Antalick G., Tempère S., Šuklje K., Blackman J.W., Deloire A., De Revel G., Schmidtke L.M. (2015). Investigation and Sensory Characterization of 1,4-Cineole: A Potential Aromatic Marker of Australian Cabernet Sauvignon Wine. J. Agric. Food Chem..

[8] Nagegowda D.A. (2010). Plant Volatile Terpenoid Metabolism: Biosynthetic Genes, Transcriptional Regulation and Subcellular Compartmentation. FEBS Lett..

[9] Reynolds A.G., Heuvel J.E. (2009). Vanden Influence of Grapevine Training Systems on Vine Growth and Fruit Composition: A Review. Am. J. Enol. Vitic..

[10] Etchebarne F., Terblanche E., Iacono M.B., Leclercq L., Angenieux M., Saurin N., Ojeda H. Minimal Pruning Increases the Concentration of Aromatic Precursors in Viognier Grapes. Proceedings of the 19èmes Journées Internationales de Viticulture GiESCO.

[11] Nan L., Liu L., Zhao X., Qiu S., Wang H., Li H. (2013). Effect of Alternative New Pruning System and Harvesting Times on Aroma Compounds of Young Wines from Ecolly (*Vitis vinifera*) in a New Grape Growing Region of the Weibei Plateau in China. Sci. Hortic..

[12] Yuxia L., Yumei G., Zhenping W. (2008). Phylogenetic Relationship Analysis of the Wine Grape Variety” Shelongzhu”. Sino-Overseas Grapevine Wine.

[13] Mateo J., Jiménez M. (2000). Monoterpenes in Grape Juice and Wines. J. Chromatogr. A.

[14] Vallee B.L., Auld D.S. (1990). Zinc Coordination, Function, and Structure of Zinc Enzymes and Other Proteins. Biochemistry.

[15] Pourali A., Afrouziyeh M., Moghaddaszadeh-ahrabi S. (2014). Extraction of Phenolic Compounds and Quantification of the Total Phenol of Grape Pomace. Eur. J. Exp. Biol..

[16] Picard M., De Revel G., Marchand S. (2017). First Identification of Three P-Menthane Lactones and Their Potential Precursor, Menthofuran, in Red Wines. Food Chem..

[17] Knupp G., Kusch P., Neugebauer M. (2002). Identification of Flavor Components in Perfumes by Headspace Solid-Phase Microextraction and Gas Chromatography-Mass Spectrometry. J. Chem. Educ..

[18] Velázquez R., Zamora E., Álvarez M.L., Hernández L.M., Ramírez M. (2015). Effects of New Torulaspora Delbrueckii Killer Yeasts on the Must Fermentation Kinetics and Aroma Compounds of White Table Wine. Front. Microbiol..

[19] Shim Y.-S., Yoon W.-J., Ha J., Seo D., Lee K.-W., Lee W.-Y., Kwon K.-I., Kang T.-S., Lee J.-H., Kim H.-J. (2013). Method Validation of 16 Types of Structural Amino Acids Using an Automated Amino Acid Analyzer. Food Sci. Biotechnol..

[20] Hashimoto K., Eckert C., Anschütz U., Scholz M., Held K., Waadt R., Reyer A., Hippler M., Becker D., Kudla J. (2012). Phosphorylation of Calcineurin B-like (CBL) Calcium Sensor Proteins by Their CBL-Interacting Protein Kinases (CIPKs) Is Required for Full Activity of CBL-CIPK Complexes toward Their Target Proteins. J. Biol. Chem..

[21] Coletta A., Toci A.T., Pati S., Ferrara G., Grieco F., Tufariello M., Crupi P. (2021). Effect of Soil Management and Training System on Negroamaro Wine Aroma. Foods.

[22] Dai Z.W., Ollat N., Gomès E., Decroocq S., Tandonnet J.-P., Bordenave L., Pieri P., Hilbert G., Kappel C., Van Leeuwen C. (2011). Ecophysiological, Genetic, and Molecular Causes of Variation in Grape Berry Weight and Composition: A Review. Am. J. Enol. Vitic..

[23] De Orduña R.M. (2010). Climate Change Associated Effects on Grape and Wine Quality and Production. Food Res. Int..

[24] Kalua C.M., Boss P.K. (2010). Comparison of Major Volatile Compounds from Riesling and Cabernet Sauvignon Grapes (*Vitis vinifera* L.) from Fruitset to Harvest. Aust. J. Grape Wine Res..

[25] Vilanova M., Genisheva Z., Bescansa L., Masa A., Oliveira J.M. (2012). Changes in Free and Bound Fractions of Aroma Compounds of Four *Vitis vinifera* Cultivars at the Last Ripening Stages. Phytochemistry.

[26] Wu Y., Zhang W., Song S., Xu W., Zhang C., Ma C., Wang L., Wang S. (2020). Evolution of Volatile Compounds during the Development of Muscat Grape ‘Shine Muscat’ (*Vitis labrusca* × *V. vinifera*). Food Chem..

[27] Rahman F.U., Nawaz M.A., Liu R., Sun L., Jiang J., Fan X., Liu C., Zhang Y. (2021). Evaluation of Volatile Aroma Compounds from Chinese Wild Grape Berries by Headspace-SPME with GC-MS. Food Sci. Technol..

[28] Slaghenaufi D., Guardini S., Tedeschi R., Ugliano M. (2019). Volatile Terpenoids, Norisoprenoids and Benzenoids as Markers of Fine Scale Vineyard Segmentation for Corvina Grapes and Wines. Food Res. Int..

[29] Drappier J., Thibon C., Rabot A., Geny-Denis L. (2019). Relationship between Wine Composition and Temperature: Impact on Bordeaux Wine Typicity in the Context of Global Warming—Review. Crit. Rev. Food Sci. Nutr..

[30] Marais J., Hunter J.J., Haasbroek P.D. (2017). Effect of Canopy Microclimate, Season and Region on Sauvignon Blanc Grape Composition and Wine Quality. S. Afr. J. Enol. Vitic..

[31] Gutiérrez-Gamboa G., Garde-Cerdán T., Rubio-Bretón P., Pérez-Álvarez E.P. (2020). Seaweed Foliar Applications at Two Dosages to Tempranillo Blanco (*Vitis vinifera* L.) Grapevines in Two Seasons: Effects on Grape and Wine Volatile Composition. Food Res. Int..

[32] Ashraf M., Foolad M.R. (2007). Roles of Glycine Betaine and Proline in Improving Plant Abiotic Stress Resistance. Environ. Exp. Bot..

[33] Kubota N., Yakushiji H., Nishiyama N., Mimura H., Shimamura K. (2001). Phenolic Contents and L-Phenylalanine Ammonia-Lyase Activity in Peach Fruit as Affected by Rootstocks. Engei Gakkai Zasshi.

[34] Hernández-Orte P., Cacho J.F., Ferreira V. (2002). Relationship between Varietal Amino Acid Profile of Grapes and Wine Aromatic Composition. Experiments with Model Solutions and Chemometric Study. J. Agric. Food Chem..

[35] Ribéreau-Gayon P., Glories Y., Maujean A., Dubourdieu D. (2021). Handbook of Enology, Volume 2: The Chemistry of Wine Stabilization and Treatments.

[36] Ardö Y. (2006). Flavour Formation by Amino Acid Catabolism. Biotechnol. Adv..

[37] Fan W., Xu Y., Jiang W., Li J. (2010). Identification and Quantification of Impact Aroma Compounds in 4 Nonfloral *Vitis vinifera* Varieties Grapes. J. Food Sci..

[38] Zhu Z., Hu K., Chen S., Xiong S., Tao Y. (2021). Increase in Fruity Ester Production during Spine Grape Wine Fermentation by Goal-Directed Amino Acid Supplementation. Fermentation.

