# Supplementary material for: Evolution of Volatile Aroma Compounds and Amino Acids in Cabernet Gernischt Grape Berries (Vitis vinifera L.): Comparison of Different Training Systems for Mechanical Soil Burial
Source: Foods. 2022 May 26;11(11):1568. doi: 10.3390/foods11111568 (PMC9180044; doi:10.3390/foods11111568)
Supplement: Supplementary file 1 [file foods-11-01568-s001.zip › foods-1696805-supplementary.pdf]

Supplementary Table S1 Basic physical and Chemical Properties of Aeolian soil

Soil type	Organic matter content/ (g·kg^{-1})	Alkaline nitrogen content/ (g·kg^{-1})	Alkaline phosphorus content/ (g·kg^{-1})	Alkaline potassium content/ (g·kg^{-1})	pH	Percentage of each grain size/%		
						Clay < 0.002 mm	Silt 0.002-0.020 mm	Sand 0.020-2.000 mm
Aeolian soil	2.74	22.35	18.64	68.44	8.2	3.45	6.11	90.44

Supplementary Table S2 Technological details of four training system

	Planting Distance(row×line)	Cluster per Vine	Cluster Weight(g)	Bunch (per cluster)	Yiled(t/ha)	Pruning Weight (Kg)
Ningxia traditional vertical trunk	3m×1m	30±2	109.82±4.77	4	8.3	2.11±0.13
Gobelet	3m×1m	25±1	124.71±6.29	4	7.4	1.92±0.11
Single Guyot	3m×1m	23±1	130.29±5.67	4	7.1	1.87±0.14
Slant Trunk with Vertical Shoot Positioning	3m×1m	24±1	133.57±9.52	4	7.3	1.54±0.15

Supplementary Table S3 Primer sequences of real-time fluorescence quantitative PCR

Gene		Sequence of primer/5′→3′	Accession number
Vv-Ecar	Forward	CGCCACAAAGTACTCTTCAAATC	JF808010
	Reverse	AATAATGCCTGGCCTCTAGC	
VvCCD1	Forward	GCTGGAGAAGCTGATAGTGAAG	NM_001280915.1
	Reverse	TGGAGAGGCTGTGAAGAATCGTGC	
Vvter	Forward	AGACTTCGCACACAGACATC	XM_002269696.1
	Reverse	CTTGCCATTGAGGTGAAACATGCCT	
VvLis	Forward	CTGTCACTTCCTCTTGTCTTCTC	AM428580.2
	Reverse	TTACACGCAACCACAACAAGCAGC	
VvActin	Forward	TCCTTGCCTTGCGTCATCTAT	EC969944
	Reverse	CACCAATCACTCTCCTGCTACAA	

Supplementary Table S4 Physical characteristics of differently training system of *Vitis vinifera* L. cv. Cabernet Gernischt berries in 2016

	50				65				70				90				120			
	CK	T1	T2	T3	CK	T1	T2	T3	CK	T1	T2	T3	CK	T1	T2	T3	CK	T1	T2	T3
Hundred-grain weight	67.71±1.85a	68.63±1.68a	58.71±3.01b	68.97±3.17a	89.51±6.06a	75.87±2.98b	64.89±3.01c	76.69±2.05b	92.47±3.52b	90.72±6.03b	107.27±3.62a	91.45±4.65b	146.45±3.48a	109.94±2.58c	132.74±4.28b	137.27±1.59b	144.43±4.28a	124.22±4.23c	132.44±2.68b	123.94±2.85c
Total soluble solids	4.61±0.38b	4.54±0.32b	4.72±0.24ab	5.22±0.3a	11.85±1.02b	14.38±1.2a	12.64±0.97ab	11.55±0.36b	14.91±0.93a	14.83±1.01a	13.06±0.9b	15.59±0.32a	16.88±0.99b	16.96±1.17b	17.4±0.56b	19.41±0.92a	21.46±1.15b	21.59±0.91b	22.92±0.96ab	24.79±1a
Titratable acids	18.47±0.49c	28.34±1.18a	24.22±0.92b	25.09±0.98b	14.07±1.18c	16.17±0.99b	19.19±1a	19.47±0.89a	8.61±1.15b	13.7±1.25a	11.68±1.38a	12.03±0.78a	5.7±0.96a	6.54±0.67a	6.88±0.67a	7.22±0.75a	5.9±0.65a	6.5±0.5a	5.57±0.49ab	4.76±0.48b
Total phenolics	8.65±0.63b	9.69±0.49a	10.34±0.51a	10.57±0.41a	5.09±0.69c	5.19±0.3c	8.89±0.21b	9.79±0.45a	4.09±0.84c	3.66±0.47c	6.18±0.66b	8.69±0.27a	3.96±0.77b	3.07±0.62b	6.45±0.4a	6.69±0.29a	2.37±0.54c	2.44±0.48c	4.14±0.38b	5.51±0.34a

Different letters in the row indicate significant differences (*p < 0.05*) among treatments for the same vintage

Supplementary Table S5 Concentrations (μg/L, mean ± SD) of volatile aroma compounds during grape development under different training system

			50				65				70				90				120			
**NO**	Compounds	Formula	CK	T1	T2	T3	CK	T1	T2	T3	CK	T1	T2	T3	CK	T1	T2	T3	CK	T1	T2	T3
	**Total acids**																					
1	Acetic acid	C2H4O2	0.11±0.9c	ND	0.51±0.28a	0.17±0.09b	0.11±0.05a	0.13 ± 0.03a	ND	0.11±0.06a	0.05±0.04b	ND	0.19±0.11a	ND	ND	0.23±0.12a	ND	0.59±0.07b	ND	0.15±0.02a	0.1 ± 0.06b	ND
2	Nonanoic acid	C9H18O2	37.46±5.47a	30.01±3.73c	26.08±2.83d	30.49 ± 3.77b	42.21 ± 3.27c	57.14 ± 8.19a	39.19 ± 5.28d	42.68 ± 6.29b	11.86 ± 1.73b	12.33 ± 3.29a	7.72±3.89c	7.19±2.81d	6.45 ± 1.98a	5.36±0.79b	5.1 ± 0.58c	4.54±0.19d	4.45 ± 1.89a	4.35±0.69c	4.36±1.78c	4.38 ± 0.48b
	**Total alcohols**																					

3	2-Pentanol	C5H12O	0.25±0.04a	0.1±0.08b	0.12±0.05c	0.16±0.02d	0.95 ± 0.34a	0.36 ± 0.09c	0.2 ± 0.11d	0.39± 0.07b	0.11± 0.03d	0.42 ± 0.12a	0.3 ± 0.11b	0.16± 0.08c	0.29 ± 0.12a	0.27± 0.07b	0.07± 0.04d	0.19± 0.09c	0.26 ± 0.11d	0.84± 0.27b	0.72± 0.22c	1.06 ± 0.39a
4	1-Penten-3-ol	C5H8O	0.19±0.04c	0.24±0.11b	ND	0.71±0.26a	ND	ND	0.93± 0.54a	0.44± 0.22b	0.2 ± 0.03b	0.1 ± 0.02c	ND	2.45± 0.84a	ND	0.4 ± 0.17c	0.45± 0.19b	0.85± 0.33a	ND	ND	0.39± 0.18a	0.77 ± 0.32b
5	Benzyl alcohol	C7H80	49±5.23b	12±3.29d	59±6.22a	33±4.07c	71 ± 4.83c	105 ± 10.04b	61 ± 3.98d	117 ± 8.9a	121 ± 8.69a	109 ± 9.28b	69 ± 7.29c	55 ± 12.82d	168 ± 17.29 b	169 ± 13.73b	136 ± 8.92c	198 ± 18.39a	157 ± 43.89c	178 ± 29.27b	106 ± 19.29d	201 ± 39.72 a
6	3-Pentanol	C5H10O	ND	ND	ND	ND	ND	ND	ND	ND	ND	0.4 ± 0.21a	ND	ND	ND	ND	ND	0.087 ± 0.063a	ND	ND	ND	0.074 ± 0.028 a
7	Isopentyl alcohol	C5H12O	ND	ND	ND	ND	ND	0.12 ± 0.1a	ND	ND	ND	ND	ND	0.05± 0.03a	ND	ND	ND	ND	ND	ND	ND	ND
8	2-Butyl alcohol	C4H10O	ND	ND	ND	ND	0.42 ± 0.33a	2.26 ± 1.21b	ND	ND	ND	0.16 ± 0.08a	2.1 ± 0.89b	ND	ND	ND	ND	0.17± 0.09a	ND	ND	0.2 ± 0.11a	0.07 ± 0.01b
9	Enanthic alcohol	C8H8O3	0.22±0.17a	ND	ND	ND	ND	ND	ND	ND	ND	ND	0.45± 0.43a	ND	ND	ND	ND	ND	0.37 ± 0.09a	ND	ND	ND
10	2-Propanol	C3H8O	ND	ND	ND	ND	ND	0.11 ± 0.07a	ND	ND	ND	ND	ND	ND	ND	ND	ND	ND	ND	0.19± 0.12a	ND	0.29 ± 0.09b

11	1-Heptanol	$C_7H_{16}O$	1.01±0.54a	1.15±0.39ab	ND	ND	ND	ND	2.32±0.89a	ND	ND	ND	ND	ND	ND	ND	ND	ND	2.99±1.34a	ND	3.1±2.02a	ND
12	Phenylethyl Alcohol	$C_8H_{10}O$	15.06±2.88a	17.08±3.39c	18.12±6.48b	24.43±5.34a	43.85±10.09b	89.92±9.98a	39.02±7.01c	32.68±6.99d	14.4±3.77c	16.36±7.29b	11.95±8.39d	37.83±7.06a	29.78±7.56b	21.82±4.28d	28.82±5.66c	47.81±7.35a	79.81±10.92a	63.18±28.78d	67.2±29.47c	69.87±11.73b
13	2-Ethyl-1-hexanol	$C_8H_{18}O$	11.87±3.76c	12.62±4.78b	5.56±5.77d	14.16±4.16a	1.89±0.77a	9.32±4.36b	5.43±2.18c	11.72±4.88a	9.89±3.65c	12.11±2.1b	12.48±3.03a	4.36±2.87d	6.55±0.93b	18.41±4.39a	5.81±1.7c	4.49±1.88d	ND	ND	ND	ND
14	(S)-3-Ethyl-4-methylpentanol	$C_8H_{18}O$	3.22±1.36a	ND	2.17±1.18b	ND	ND	ND	ND	1.94±0.54a	ND	ND	ND	ND	ND	ND	ND	ND	ND	ND	ND	ND
15	1,2-Dihydrolinalool	$C_{10}H_{20}O$	ND	2.57±0.89a	ND	ND	ND	ND	ND	ND	ND	ND	ND	ND	ND	ND	ND	ND	ND	ND	ND	ND
16	2-Ethoxy-ethanol	$C_4H_{10}O_2$	ND	ND	ND	ND	ND	ND	2.56±0.49a	3.91±1.32b	2.56±0.8a	ND	ND	2.94±1.02ab	3.26±1.18a	ND	3.51±0.42a	ND	ND	ND	ND	ND
17	4-Methyl-1-hexanol	$C_7H_{16}O$	ND	ND	ND	ND	2.78±0.62a	ND	ND	2.42±1.51ab	2.17±1.27d	2.4±0.83a	2.27±1.38b	2.21±0.79c	ND	2.84±2.21a	2.61±1.78a	ND	2.57±0.64a	ND	ND	2.45±0.72a
18	α-Methyl-benzenemethanol	$C_8H_{10}O$	ND	ND	ND	ND	ND	ND	ND	ND	ND	ND	ND	ND	ND	ND	ND	ND	8.56±3.28a	ND	6.3±1.81b	5.93±2.84c
19	1-Phenyl-1-decanol	$C_{16}H_{26}O$	ND	ND	ND	ND	ND	ND	ND	ND	ND	ND	ND	ND	4.07±3.22a	ND	ND	ND	ND	3.86±1.47a	ND	4.34±1.38b

	**Total carbonyl compounds**																					
21	Octanal	$C_8H_{16}O$	0.01±0.01a	ND	ND	0.05±0.02b	0.94 ± 0.28a	1.46 ± 0.32b	ND	ND	0.21± 0.12a	0.27 ± 0.07a	ND	ND	0.27 ± 0.08a	ND	ND	0.22± 0.11a	ND	0.87± 0.23a	0.49± 0.18b	0.88 ± 0.43a
22	Acrolein	C_3H_4O	0.12±0.03a	ND	ND	ND	ND	0.1±0.1a	ND	ND	0.04± 0.02a	0.04 ± 0.02a	ND	ND	0.04 ± 0.03a	ND	ND	ND	ND	ND	0.15± 0.07a	ND
23	Trans-2-pentenal	$C_8H_{14}O$	ND	20.15±3.83a	ND	ND	27.5 ± 5.33d	46.82 ± 7.48a	35.33 ± 4.29c	41.4± 9.02b	33.5± 6.3c	45.11 ± 4.39a	36.33 ± 9.25b	40.35 ± 10.21a	34.74 ± 5.22a	31.29 ± 4.32c	27.68 ±8.1d	32.15 ± 8.33b	19.95 ± 7.29d	57.11 ± 22.19a	28.69 ± 9.32c	39.07 ± 10.06b
24	4-Cis-decenal	$C_{10}H_{18}O$	ND	ND	ND	ND	ND	ND	ND	ND	ND	ND	ND	ND	0.91 ± 0.65a	ND	ND	ND	ND	ND	ND	ND
25	1,3-Cyclopentadione	$C_5H_6O_2$	0.16±0.06b	0.21±0.11a	0.17±0.03b	0.11±0.02c	ND	ND	0.07± 0.04a	0.1 ± 0.03ab	1.14± 0.45b	ND	0.3 ± 0.1c	1.83± 0.56a	1.03 ± 0.28b	0.87± 0.19c	ND	2.12± 1.22a	ND	0.14± 0.05b	0.13± 0.04b	0.25 ± 0.11a
26	Artemisia ketone	$C_{10}H_{16}O$	0.17±0.08a	0.38±0.13b	0.77±0.46c	ND	0.1 ± 0.06c	0.2 ± 0.12a	0.13± 0.09b	0.04± 0.01d	0.06± 0.02a	0.06 ± 0.03a	ND	ND	0.03 ± 0.02a	0.03± 0.01a	0.08± 0.04b	ND	0.03 ± 0.01a	0.02± 0.01a	0.03± 0.01a	0.04 ± 0.02a
27	Benzaldehyde	C_7H_6O	9.06±3.43b	ND	5.93±2.28c	9.84±4.29a	8.86 ± 3.98d	21.56 ± 5.3a	16.39 ± 3.93c	17.51 ± 4.72b	13.25 ± 4.89d	20.36 ± 3.19a	17.02 ± 5.33c	18.72 ± 2.81b	20.3 ± 9.19a	15.56 ± 3.29b	15.09 ± 4.42c	8.36± 1.92d	15.79 ± 4.38a	15.23 ± 4.11b	9.71± 0.91d	15.01 ± 3.16c
28	2,6-	$C_9H_{10}O$	20.75 ±	21.76±2.1a	20.63±3.89c	19.39 ±	14.22 ±	ND	16.5±	18.65	19 ±	ND	21.5±	24.94	21.16	21.12	22.04	18.99	23.49	21.34	22.48	20.32

	Dimethylbenzaldehyde		4.18b			3.11d	5.22c		2.91b	± 7.73a	3.21c		8.29b	± 3.02a	± 2.19b	± 4.22c	± 1.18a	± 6.93d	± 2.85a	± 6.28c	± 4.19b	± 1.03d
29	2-Methyl-benzaldehyde	C8H8O	84.75±8.22c	96.05±3.59a	79.05±2.17d	85.1±7.48b	96.53 ± 3.13c	70.92 ± 6.53d	117.86 ± 3.21b	119.97 ± 8.43a	106.39 ± 4.26c	114.06 ± 3.11a	111.86 ± 6.53b	ND	119.59 ± 8.74a	ND	115.41 ± 12.19b	90.06 ± 8.92c	121.77 ± 14.23b	110.42 ± 8.79d	121.79 ± 22.17a	114.45 ± 10.01c
30	(E,E)-2,4-Hexadienal	C6H8O	17.89±4.89c	20.98±3.21b	23.13±5.28a	23.98 ± 7.31a	18.53 ± 4.28a	23.87 ± 12.73c	25.13 ± 3.54b	28.24 ± 5.21a	18.9± 4.27d	28.38 ± 3.19b	27.29 ± 9.02c	42.37 ± 6.48a	19.59 ± 4.89a	37.9± 10.24b	30.89 ± 5.18c	45.89 ± 8.48a	37.58 ± 6.32a	45.65 ± 9.49b	33.21 ± 11.28c	54.2 ± 7.33a
31	(E)-2-Heptenal	C7H12O	ND	ND	ND	ND	ND	ND	ND	ND	ND	ND	ND	ND	ND	ND	ND	ND	5.91 ± 1.07a	ND	ND	ND
32	Acetone	C3H60	ND	2.32±0.72a	3.11±1.21b	ND	3.01 ± 2.81a	2.61 ± 1.23d	2.91± 0.75b	2.81± 1.18c	2.86± 0.83a	ND	2.67± 1.24b	2.3 ± 0.92c	2.42 ± 1.28c	2.52± 1.12b	2.59± 0.89a	2.42± 0.42c	1.78 ± 0.63d	2.41± 0.28c	2.65± 1.27b	2.82 ± 0.95a
33	Phenyl methyl ketone	C8H8O	ND	ND	ND	ND	ND	ND	ND	ND	ND	16.23 ± 3.82a	ND	ND	ND	23.8± 5.23a	ND	ND	ND	25.88 ± 6.28a	ND	19.19 ± 3.38b
	**Total benzenes**																					
34	1,4-dimethoxy-Benzene	C8H10O2	ND	9.31±2.75a	7.92±1.92b	9.4±3.24a	10.03 ± 4.23c	9.35 ± 2.19d	12.02 ± 5.33b	12.59 ± 1.25a	11.61 ± 1.74d	13.97 ± 4.22a	12.54 ± 1.91c	13.31 ± 4.03b	14.33 ± 3.37a	12.49 ± 4.28c	12.61 ± 2.91b	10.77 ± 3.95d	13.3 ± 4.26b	12.03 ± 1.78d	14.34 ± 2.09a	12.77 ± 3.91c
35	2-Methoxy-1,3,5-trimethyl-Benzene	C10H14O	ND	5.8±1.02a	ND	ND	5.95 ± 0.83c	6.33 ± 2.71b	5.99± 1.82c	6.61± 4.31a	6.73± 2.18c	8.13 ± 1.79a	7.99± 2.16b	8.06± 3.64a	8.47 ± 3.93a	6.13± 2.17c	7.77± 0.89b	ND	ND	ND	7.8 ± 3.38a	ND

36	1,3-Dimethyl benzene	C8H10	ND	ND	2.46±2.21a	ND	ND	ND	ND	ND	ND	ND	ND	ND	ND	ND	ND	ND	ND	ND	ND	ND
37	1,2,3,4-tetrahydro-1,1,6-trimethyl-Naphthalene	C13H18	ND	ND	ND	ND	ND	ND	ND	ND	ND	ND	ND	ND	ND	ND	ND	ND	ND	30.99 ± 4.28c	74.28 ± 8.21a	32.13 ± 6.39b
	**Total C6/C9 Compounds**																					
38	1-Hexanol	C6H14O	ND	ND	0.56±0.43a	1.5±1.1b	1.37 ± 0.47a	0.59 ± 0.33b	0.31±0.17d	0.47±0.23c	21.91 ± 10.08a	ND	ND	54.83 ± 22.72b	43.04 ± 14.72c	22.6±8.27d	98.93 ± 12.29b	100.1 ± 21.25a	69.71 ± 8.45c	63.85 ± 9.92d	126.47 ± 30.09b	280.58 ± 29.93a
39	Isohexyl alcohol	C6H14O	0.45±0.22c	0.24±0.17d	1.6±0.67a	0.83±0.32b	2.65 ± 0.94b	0.52 ± 0.17c	ND	3.75±1.21a	ND	1.51 ± 0.78b	0.73±0.17c	3.17±1.87a	0.11±0.04c	0.36±0.21b	1.85±0.71a	0.2 ± 0.2c	1.97 ± 0.45a	0.89±0.72d	1.53±1.18c	1.88 ± 0.65b
40	4-Hexen-1-ol	C6H12O	ND	ND	1.6±0.6a	9.3±4.27b	0.47 ± 0.16b	0.33 ± 0.22c	ND	1.21±0.48a	0.21±0.12b	0.19 ± 0.04c	0.69±0.24a	ND	0.68 ± 0.38b	ND	0.36±0.16c	0.98±0.27a	ND	0.08±0.03a	ND	0.88 ± 0.43b
41	2,3-Hexanediol	C6H14O2	ND	ND	0.29±0.11a	2.94±1.13b	0.13 ± 0.03a	0.29 ± 0.12b	ND	ND	ND	ND	0.61±0.32a	0.69±0.28ab	ND	0.26±0.09a	ND	0.5 ± 0.27b	6.3 ± 2.22a	6 ± 1.87b	0.55±0.28c	0.44 ± 0.12c
42	Cis-3-Hexenol	C6H14O	ND	ND	ND	ND	ND	ND	ND	ND	18.46 ± 4.92b	22.84 ± 7.81a	12.07 ± 2.02c	11.91 ± 3.76d	16.15 ± 4.02b	28.15 ± 3.22a	12.99 ± 3.83c	10.51 ± 1.98d	16.46	78.15	10.37	14.18
43	Trans-2-hexenol	C6H14O	ND	ND	ND	ND	ND	ND	ND	ND	31.73 ± 10.03a	ND	ND	ND	33.18 ± 4.82a	45.28 ± 8.92b	ND	ND	32.38 ± 10.83a	ND	41.99 ± 2.34b	ND

44	Cis-2-hexenol	C6H12O	ND	ND	ND	ND	0.15 ± 0.07a	ND	ND	ND	ND	ND	8.2 ± 3.1a	ND	ND	1.5 ± 0.65a	0.86± 0.29b	ND	10.71 ± 2.17a	8.79± 1.81b	ND	6.11 ± 1.93c
45	Methyl formate	C9H18O2	0.35±0.17d	0.64±0.29b	1.05±0.43a	0.49±0.18c	0.21 ± 0.11d	0.39 ± 0.17b	0.3 ± 0.21c	0.55± 0.2a	0.45± 0.32a	0.44 ± 0.18a	0.11± 0.1c	0.42± 0.13b	0.19 ± 0.11d	0.29± 0.04b	0.31± 0.11a	0.25± 0.08c	1.53 ± 0.43a	0.42± 0.32d	1.11± 0.61b	0.99 ± 0.27c
46	Ethyl formate	C3H6O2	0.4±0.1b	0.27±0.12b	0.4±0.2b	1.03±0.34a	0.24 ± 0.11c	0.22 ± 0.04d	0.55± 0.17b	0.83± 0.2a	0.23± 0.04d	0.48 ± 0.12c	0.88± 0.18a	0.81± 0.23b	0.29 ± 0.16c	0.54± 0.21a	0.53± 0.11a	0.35± 0.1b	1.99 ± 0.67a	0.48± 0.2d	0.71± 0.13c	1.38 ± 0.68b
47	Propynyl propionate	C6H8O2	0.06±0.01c	0.64±0.23a	ND	0.19±0.06b	0.13 ± 0.02c	0.26 ± 0.11b	ND	1.11± 0.38a	ND	0.17 ± 0.05c	0.38± 0.14a	0.24± 0.07b	0.26 ± 0.12d	0.35± 0.23c	1.69± 0.35a	0.45± 0.19b	0.21 ± 0.03c	ND	1.1 ± 0.54a	0.42 ± 0.27b
48	Melonal	C9H16O	0.4±0.13b	0.31±0.05b	0.1±0.03c	0.54±0.23a	0.39 ± 0.17c	0.44 ± 0.1b	0.35± 0.11d	0.85± 0.09a	0.86± 0.23b	0.88 ± 0.14b	1.84± 0.42a	1.9 ± 0.51a	1.02 ± 0.29a	1.33± 0.41c	1.99± 0.81b	2.67± 1.21a	1.2 ± 0.2c	1.25± 0.41c	2.49± 1.02b	3.5 ± 1.25a
49	Hexanal	C6H12O	0.11±0.03d	0.44±0.12a	0.25±0.05c	0.39±0.13b	0.23 ± 0.02d	37.85 ± 4.02c	41.04 ± 3.98b	56.68 ± 10.37a	61.18 ± 6.67a	88.89 ± 14.83 b	61.99 ± 9.28c	118.87 ± 21.17a	65.89 ± 17.24 d	128.48 ± 30.04b	130.71 ± 17.16a	123.39 ± 13.28c	86.53 ± 7.38d	361.69 ± 40.59a	152.72 ± 32.19b	137.8 ± 18.82 c
50	2-Hexenal	C6H10O	336.33 ± 43.29c	492.9 ± 73.29b	326.5±38.19d	497.43 ± 47.41a	1102.15 ± 210.26d	2020.13 ± 173.19a	1552.7 4 ± 197.28 c	1854.7 3 ± 402.16 b	1586.1 2 ± 327.17 d	2112.0 8 ± 272.11 a	1715.5 7 ± 421.9c	1897.3 4 ± 290.01 b	1617. 8 ± 442.8 7a	1546.3 1 ± 289.17 b	1390.4 8 ± 510.23 d	1514.1 9 ± 179.51 c	1089. 1 ± 119.04 d	1622.7 2 ± 277.87 b	1367.9 9 ± 421.72 c	1803. 74 ± 290.3 7a
51	2-Nonanone	C9H18O2	ND	0.23±0.05a	0.06±0.02b	ND	ND	0.45 ± 0.13a	ND	0.25± 0.11b	1.27± 0.43a	0.83 ±	1.09± 0.29b	0.32± 0.07d	1.37 ±	1.37± 0.51a	0.95± 0.23b	0.55± 0.19c	1.13 ±	1.32± 0.61c	1.84± 0.73a	1.77 ±

											0.11c			0.24a				0.28d			0.39b
52	2H-Pyran-2-methanol	C6H12O2	39.53±5.39a	31.57±2.99b	24.19±7.52d	30.54 ± 4.47c	ND	ND	ND	ND	ND	ND	ND	ND	ND	ND	ND	ND	ND	ND	ND	ND
53	D，L-Erythro-1-phenyl-1,2-propanediol	C9H12O2	ND	ND	ND	ND	ND	ND	ND	6.19±2.73a	ND	ND	4.52±1.71a	4.83±0.95a	ND	4.78±2.21a	3.91±1.73b	2.5 ± 0.59c	4.15 ± 2.77a	7.3 ± 4.18a	3.78±1.09c	ND
	**Total esters**																					
54	Propiolic acid ethyl ester	C5H6O2	0.14±0.06b	0.14±0.04b	ND	0.37±0.11a	ND	ND	0.4 ± 0.17a	1.03±0.31b	0.33±0.07c	0.35 ± 0.21b	ND	1.39±0.72a	0.37 ± 0.11c	0.31±0.08c	2.05±1.12b	3 ± 0.87a	0.32 ± 0.04a	1.31±0.47c	15.43 ± 2.77b	19.89 ± 4.23a
55	Phenylethyl phenylacetate	C16H16O2	0.83±0.23a	ND	ND	1.86±0.56b	4.12 ± 1.71c	ND	17.08 ± 4.97a	5.32±1.31b	0.5 ± 0.2c	0.25 ± 0.13d	0.79±0.31b	1.7 ± 0.23a	0.21 ± 0.02d	0.25±0.12c	0.78±0.21a	0.29±0.13b	0.34 ± 0.2d	1.47±0.45c	2.09±1.13b	2.98 ± 1.51a
56	Ethyl lactate	C5H10O3	0.84±0.12a	0.8±0.41a	0.78±0.26a	0.66±0.17b	0.36 ± 0.09a	0.15 ± 0.03c	0.23±0.11b	ND	ND	0.44 ± 0.17a	ND	5.34±1.73b	0.7 ± 0.26a	ND	ND	0.18±0.04b	ND	0.51±0.19a	ND	0.22 ± 0.07b
57	2-Methyl butynoate	C5H6O2	ND	1.53±0.23a	1.33±0.48b	1.02±0.32c	1.54 ± 0.41b	0.26 ± 0.13d	0.36±0.14c	2.28±1.04a	0.6 ± 0.15b	2.25 ± 1.82a	0.69±0.19b	0.33±0.1c	0.2 ± 0.1c	0.23±0.11c	0.42±0.27b	0.77±0.33a	0.58 ± 0.34b	0.16±0.04a	ND	0.64 ± 0.23a
58	Methyl salicylate	C8H8O3	12.53±4.27a	12.14±3.93c	9.87±1.82d	12.23 ± 3.19b	13.98 ± 4.21d	15.54 ± 2.09c	15.87 ± 3.85b	16.61 ± 4.27a	12.32 ± 4.04d	16.48 ± 3.73a	14.41 ± 4.89b	14.09 ± 3.71c	14.67 ± 5.43a	12.27 ± 3.77c	13.37 ± 2.15b	10.43 ± 1.99d	13.29 ± 5.65a	11.71 ± 3.08c	11.85 ± 4.92b	11.52 ± 1.96d
	**Total norisoprenoids**																					
59	β-Damascenone	C13H18O	85.12 ± 20.12a	ND	65.11±17.43b	62.34 ± 12.88c	82.36 ± 24.25a	106.76 ± 37.81b	86.69 ±	107.9 ±	84.07 ±	107.68 ±	122.43 ±	129.11 ±	106.16 ±	76.18 ±	90.09 ±	91.46 ±	85.31 ±	122.07 ±	130.34 ±	103.96 ±

									22.18c	41.09a	13.71a	27.83c	31.11b	13.29a	38.52a	24.05d	19.7c	13.29b	25.93 d	27.01b	34.79a	25.8c
60	β-Ionone	C13H20O	ND	ND	ND	ND	ND	ND	ND	ND	ND	ND	ND	ND	5.12 ± 2.17a	ND	2.22±0.54c	4.39±1.38b	5.22 ± 1.52b	3.33±0.78c	5.37±2.4a	ND
	**Total terpenoids**																					
61	Linalool	C10H18O	ND	ND	ND	ND	ND	ND	ND	ND	ND	ND	ND	ND	ND	1.82±0.39a	1.89±0.47a	ND	ND	1.48±0.36b	2.84±0.78a	ND
62	α-Terpineol	C10H18O	3.4±1.23a	ND	ND	ND	ND	ND	ND	ND	3.57±0.69a	ND	2.56±1.54c	4.14±1.31a	ND	ND	2.88±1.3a	ND	8.9 ±3.32a	5.74±1.51b	5.25±1.42c	5.21 ± 0.79c
63	β-Citronellol	C10H20O	ND	ND	ND	ND	ND	ND	ND	ND	0.3 ± 0.1b	ND	0.73±0.22a	0.68±0.13a	0.34 ± 0.14c	0.94±0.36a	0.93±0.21a	0.89±0.52b	0.84 ± 0.32c	0.99±0.3b	1.28±0.71a	1.2±0.11a
64	Geraniol	C10H18O	ND	ND	ND	ND	ND	ND	ND	ND	ND	ND	ND	0.019 ± 0.01a	ND	0.29±0.2a	0.19±0.1b	ND	0.018 ± 0.018 b	0.28±0.12a	0.29±0.13a	ND
65	Myrcene	C10H16	ND	ND	ND	0.019 ± 0.012a	ND	ND	ND	ND	0.019 ± 0.013a	ND	ND	ND	ND	ND	0.029 ± 0.02a	0.039 ± 0.03b	0.029 ± 0.021c	0.038 ± 0.025c	0.088 ± 0.043b	0.19 ± 0.06a
	**Total volatile phenols**																					
66	Phenol, 3,5-bis(1,1-dimethylethyl)-	C14H22O	7.47±3.21a	6.19±2.87b	ND	ND	ND	8.14 ± 3.79a	ND	ND	ND	9.43 ± 4.27a	ND	10.31 ± 3.92b	9.69 ± 2.84a	ND	ND	8.76±4.93b	ND	ND	ND	8.48 ± 4.22a

67	2,4-Di-tert-butylphenol	C14H22O	ND	ND	ND	6.25±1.42a	ND	ND	ND	2.92±0.79a	7.14±5.48a	ND	10.52±3.11b	ND	ND	ND	11.5±6.29a	ND	ND	ND	6.73±0.8a	ND
	**Others**																					
68	2-Ethylfuran	C6H8O	ND	0.48±0.21a	ND	ND	ND	0.4±0.11a	ND	ND	ND	ND	ND	1.35±0.54a	ND	ND	ND	0.43±0.32a	ND	ND	ND	ND
69	Cyclohexasiloxane, dodecamethyl-	C12H36O6Si6	4.72±1.67b	3.56±1.24c	4.98±3.51a	3.03±1.02d	ND	3.01±1.28c	5.68±2.74b	6.2±3.27a	12.56±4.38b	11.61±3.29c	7.37±3.09d	19.21±3.81a	10.69±4.29c	12.03±6.33b	16.11±2.95a	6.03±4.27d	13.08±4.74d	15.22±6.35b	10.3±2.14c	17.7±6.46a
70	2-Propylfuran	C7H10O	1.05±0.44d	3.02±2.87c	3.69±1.58a	3.31±1.21b	3.02±2.91a	ND	2.48±1.32b	ND	ND	ND	3.27±1.24a	2.85±1.39b	1.94±0.54a	ND	ND	ND	ND	ND	ND	ND

[1]Tukey's HSD test, different letters indicate significant differences at $P < 0.05$.

[2] ND: not detected.
